# Tissue adhesives for wound closure

**DOI:** 10.1002/SMMD.20220033

**Published:** 2023-02-12

**Authors:** Bin Kong, Cheng Qi, Huan Wang, Tiantian Kong, Zhou Liu

**Affiliations:** ^1^ Department of Biomedical Engineering School of Medicine Shenzhen University Shenzhen Guangdong China; ^2^ College of Mechatronics and Control Engineering Shenzhen University Shenzhen Guangdong China; ^3^ The Eighth Affiliated Hospital Sun Yat‐Sen University Shenzhen China; ^4^ Department of Urology Institute for Translational Medicine The First Affiliated Hospital of Shenzhen University Shenzhen Second People's Hospital Shenzhen Guangdong China; ^5^ College of Chemistry and Environmental Engineering Shenzhen University Shenzhen Guangdong China

**Keywords:** adhesive, cornea, gastrointestinal tissues, materials, skin, wound

## Abstract

Tissue adhesives have raised much attention from scientists in recent years. They have been extensively utilized in various medical fields, such as wound closure, due to the advantages of being simple, time‐saving, and avoiding the problems and complications associated with surgical sutures. Besides, the tissue adhesives can absorb wound exudates and promote tissue repair. The rapid evolution in the field of tissue adhesives has resulted in the development of various adhesives with excellent mechanical properties and superior functions. However, many challenges still restrict their use in numerous clinical applications. In this paper, we present an up‐to‐date review of tissue adhesives for wound closure. We mainly discussed the fundamental design requirements for the adhesives, the fabrication of tissue adhesives, and the application of tissue adhesives on skin healing, corneal patch, and gastrointestinal tissues. We then highlighted the current challenges and unmet needs and delineated potential new clinical development directions for future adhesives. The progress in tissue adhesives will provide novel approaches for wound management and has the potential to supply effective treatments for a variety of medical applications.

## Highlights

- The tissue adhesives for wound closure are comprehensively reviewed.
- Focus on the discussion of design requirements, fabrication, and application of tissue adhesives on skin healing, corneal patch, and gastrointestinal tissues.
- Summarize the challenges for the adhesives and future perspectives.

## Graphical abstract

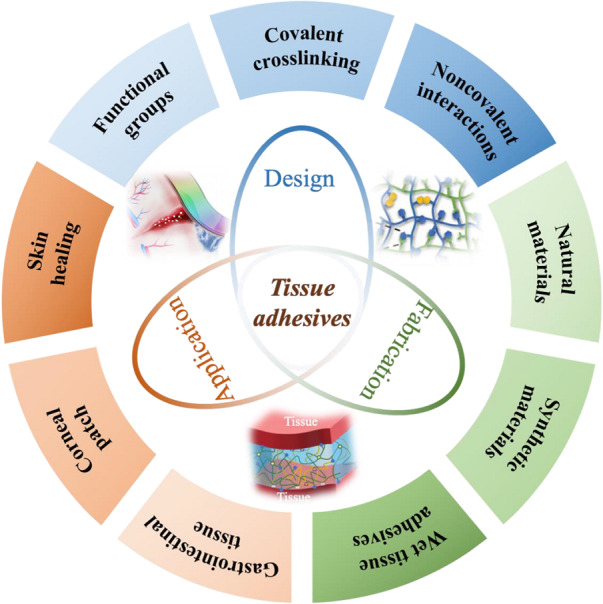

## 1 INTRODUCTION

Wounds are usually triggered by external forces, which will subsequently damage the structure of cells, blood vessels, and extracellular matrix (ECM).^1^, ^2^ To avoid infection, any tissue damage needs to be closed and repaired in a timely manner.^3^, ^4^, ^5^ Surgical sutures are widely used to seal and repair tissues since they have high tensile strength and low dehiscence rate, facilitating wound closure.^6^, ^7^ However, the surgical suturing process is inherently damaging to the tissues, may require anesthesia, and has a high probability of postoperative infection, inflammation, nerve damage, and scar tissue formation.^8^, ^9^ In addition, the surgical suturing process is time‐consuming and requires a high level of suturing skills for certain specific tissues, which affects the success rate of the procedure.^10^, ^11^ In recent years, tissue adhesives have become a promising alternative to sutures, with the advantages of being simple, time‐saving, and avoiding the problems and complications associated with surgical sutures, and have therefore received much attention and research.^12^, ^13^, ^14^

Tissue adhesives have been extensively employed as wound dressing for rapid tissue wound treatment and inductive regeneration.^15^, ^16^ Due to the complexity of the wound, tissue adhesives should be biocompatible and biodegradable to allow proper tissue repair and have strong adhesive strength to the tissues, mechanical stability for bearing the dynamic force from tissues, and low cost of production.^17^, ^18^ Nevertheless, preparing a tissue adhesive that can fulfill all requirements is still tricky. Suitable tissue adhesives should be developed according to the properties required for a particular application and the nature of the wounds.^19^, ^20^, ^21^ The successful preparation of adhesives involves an interdisciplinary effort, such as the chemical, mechanical and biological intersections, since the physicochemical abilities of the adhesives mainly dictate the properties of the adhesives, the interactions between the tissue and adhesives, the immunological responses of the host, and the topical environmental features.^22^, ^23^, ^24^ To date, although researchers have produced and commercialized a variety of tissue adhesives, many challenges still restrict their use in numerous clinical applications.

**FIGURE 1:**
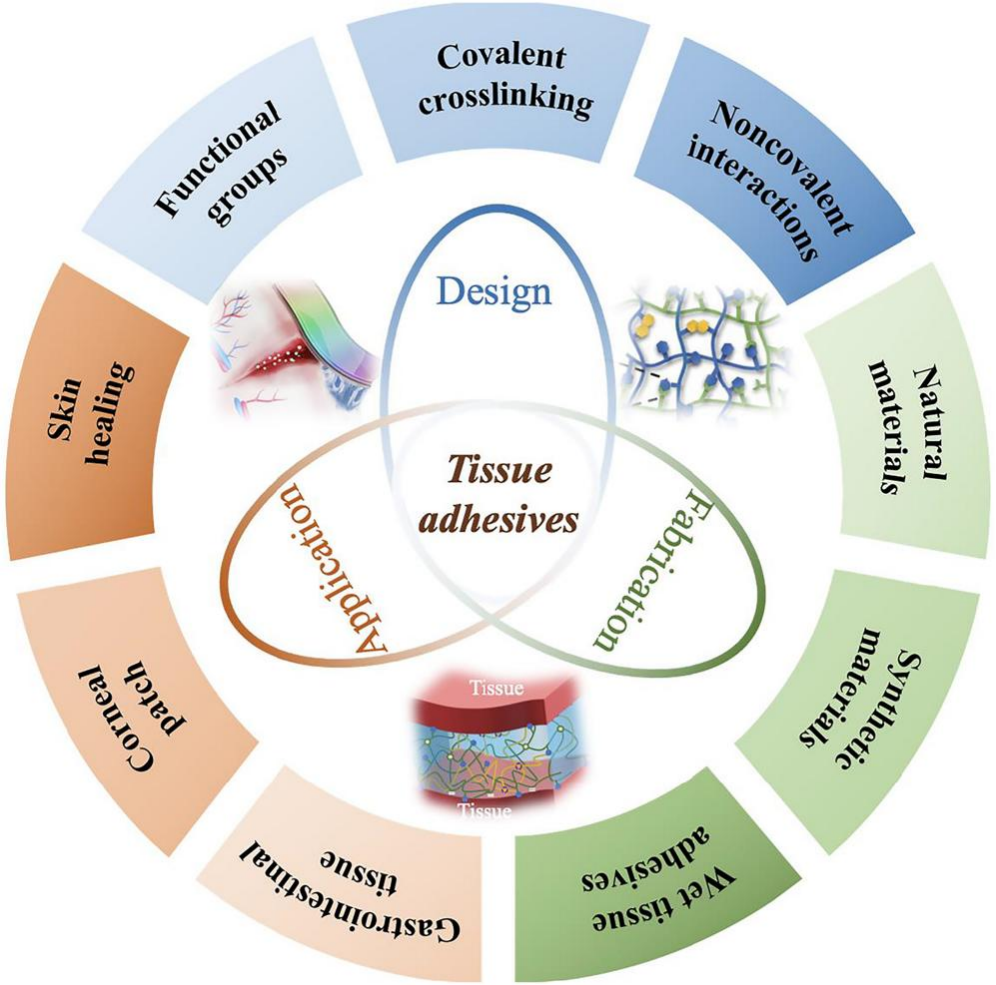
The schematic diagram of tissue adhesives for wound closure.

In this review, we present an up‐to‐date review of the tissue adhesives for wound closure (Figure 1). We first discussed the fundamental design requirements for the adhesives concisely. The nature of tissues and the functional chemical groups on the tissue surface was introduced, followed by the discussion of the most widely used reactive groups for the adhesives. After that, we introduced the fabrication and recent significant advances of several tissue adhesives, including synthetic and natural materials‐derived adhesives. Then, we mainly introduced the application of tissue adhesives on skin healing, corneal patch, and gastrointestinal tissues with examples that have achieved higher performances and advanced functionalities. Finally, we highlighted the current challenges and unmet needs and delineated potential new clinical development directions for future adhesives.

## 2 DESIGN REQUIREMENTS FOR THE ADHESIVES

The tissue consists of liquid and solid components, where the solid ingredients are widely used for adhesion. Proteins are the essential human body element that can provide mechanical support to ECM, such as the interconnected protein filaments in the cytoskeleton, glycoproteins in the cell membrane, and fibronectin in the ECM.^25^ Functional chemical groups of the tissues can covalently interact with tissue adhesive, which mainly originates from the amino acid residues of the protein, such as amine, carboxylate, and thiol group.^3^ Lysine has a positively charged residue, which can offer primary amines, while glutamic acid, as acidic amino acid, can provide carboxylic acids.^26^ Besides, cysteine contributes to the presentation of the thiol group, but its concentration is generally low. Among these groups, primary amines are the most extensively utilized ones in the adhesive strategy since they are abundant and chemically reactive.^19^, ^27^, ^28^ Additional constituents of the ECM can be a target as well. For instance, fatty acids, terminated with carboxylic acids, can allow firm adhesion to the tissue.^29^

**FIGURE 2:**
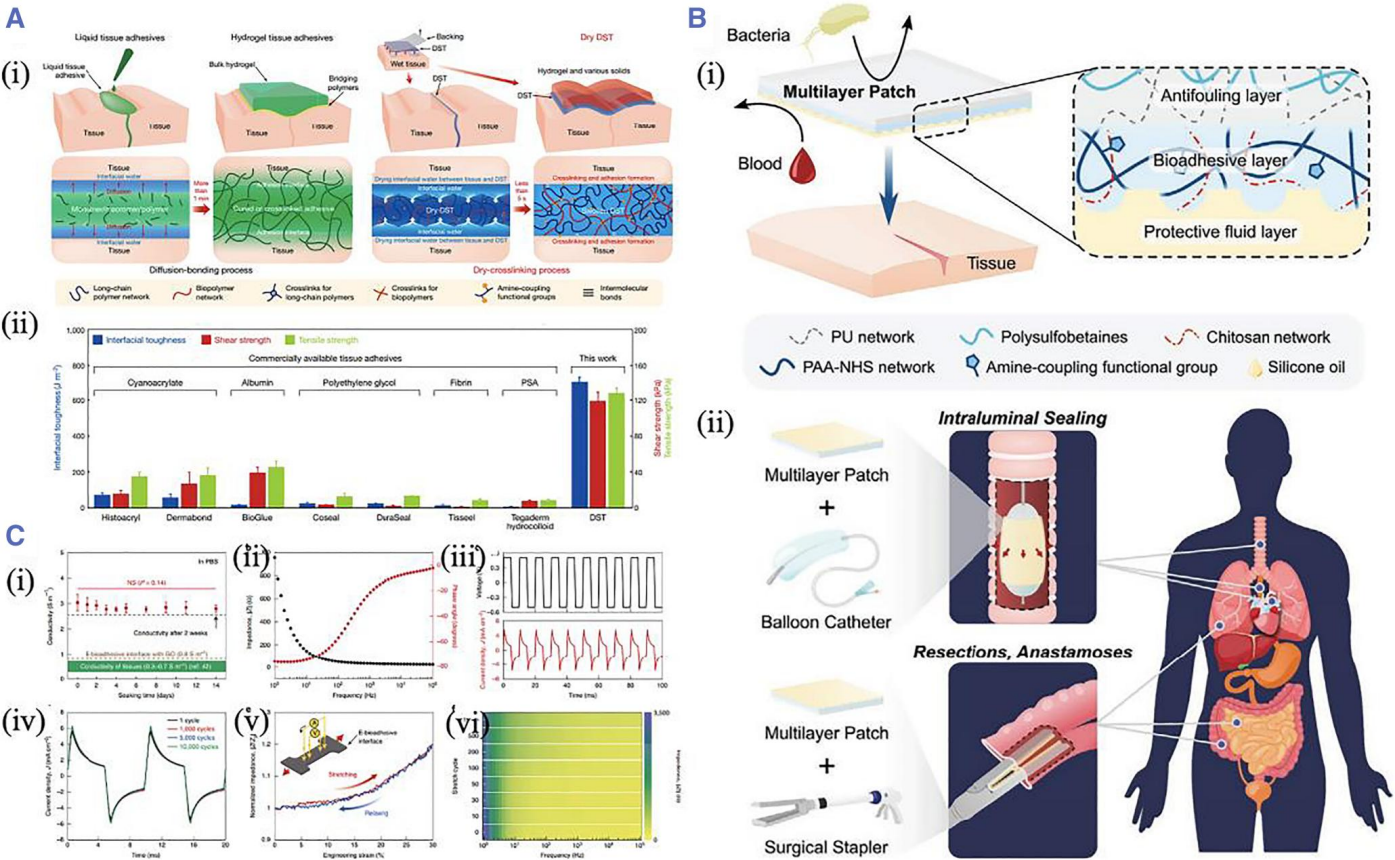
(A) (i) Scheme showing the fabrication of DST hydrogel and its mechanism of adhesion on wet tissues; (ii) The shear strength, tensile strength, and interfacial of the DST hydrogel and other commercialized adhesives on the porcine skin. Reproduced with permission.^36^ Copyright 2019, The Authors, published by Springer Nature. (B) Schematic illustration showing (i) the multilayer composition of the adhesive patch; (ii) the minimally invasive surgical application of the multilayer patch. Reproduced with permission.^37^ Copyright 2021, John Wiley and Sons. (C) (i) Surface conductivity of the bioadhesive device. (ii) Electrical impedance spectroscopy measurement. (iii) Charge injection curve. (iv) Charge injection curve at different cycles. (v, vi) Impedance in the thickness direction of the bioadhesive. Reproduced with permission.^38^ Copyright 2021, The Authors, published by Springer Nature. DST, double‐sided tissue.

Most current and burgeoning tissue adhesives employ a variety of reactive groups for covalent crosslinking with these functional groups on the tissues.^30^, ^31^, ^32^ N‐hydroxysuccinimide (NHS) ester is a reactive acylating agent often applied in biological chemistry.^33^, ^34^, ^35^ They react quickly with primary amines under physiological or mildly basic environments, forming amide bonds by releasing NHS leaving moiety. Zhao's group presented a dry double‐sided tissue (DST) adhesive composed of biopolymer and polyacrylic acid grafted with NHS. The results from the in vitro and in vivo animal models demonstrated that DST could obtain robust adhesion on a variety of wet tissues within 5 s^36^ (Figure 2A). Further, they constructed a multilayer bioadhesive patch to seal tissues with minimal invasion. The patch could reject fluid from the body and develop a rapid, pressure‐triggered adhesion to wet tissue with anti‐biofouling and anti‐inflammatory properties. Among the three layers, the pressure‐triggered adhesive layer was mainly fabricated by the acrylic acid, chitosan, and acrylic acid NHS to form PAAc‐NHS hydrogel, which could adhere tightly to the tissues through the covalent crosslinking with the amines in the tissue^37^ (Figure 2B). Besides, based on the previous study, they reported an electronic‐bioadhesive interface based on thin‐layer graphene nanocomposites that enabled bioelectronic devices to provide fast, robust, and removable‐on‐demand integration on different moist dynamic tissues. The high electrical conductivity at the interface offered the possibility of bi‐directional bioelectronic communication^38^ (Figure 2C). Except for amine, NHS ester can generate thioester by reacting with thiol. However, this product readily underwent degradation through hydrolysis or exchange with the adjacent amine in a transamidation reaction.

**FIGURE 3:**
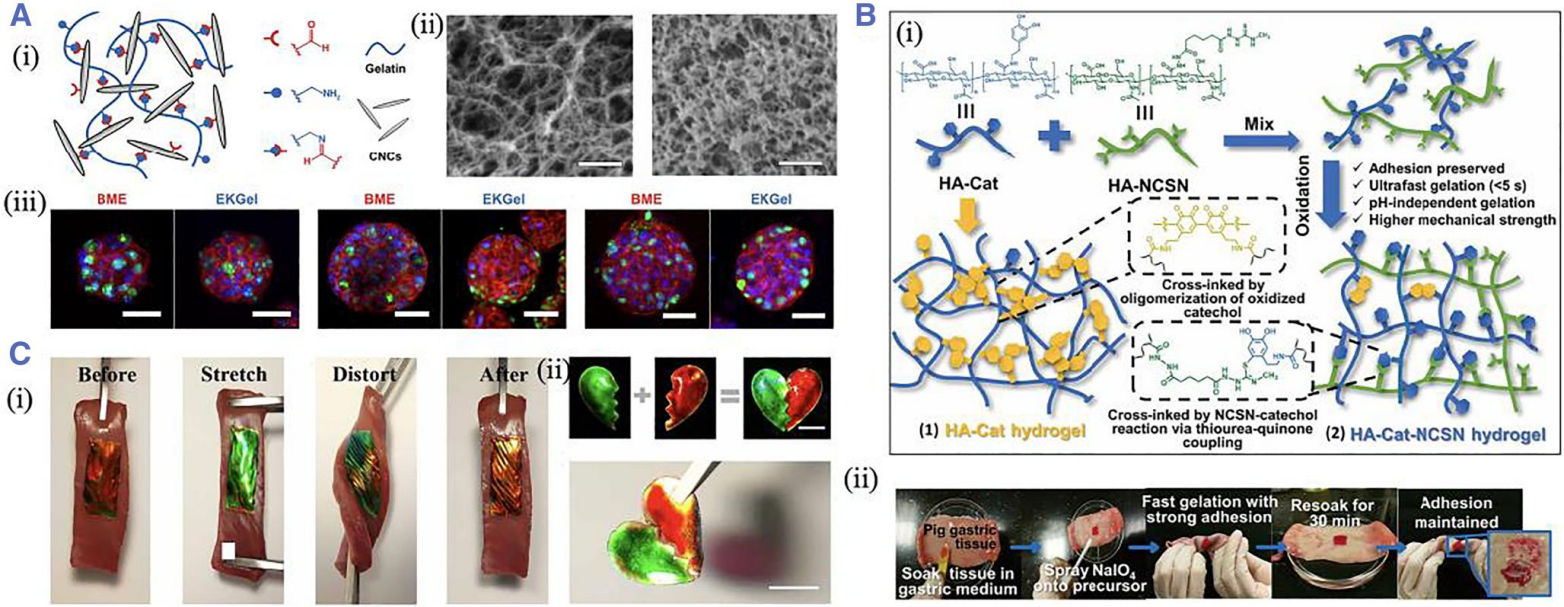
(A) (i) Scheme showing the crosslinking mechanism of EKGel; (ii) The SEM images of the EKGel. (iii) The organoid formation in the EKGel. Reproduced under terms of the CC‐BY license.^44^ Copyright 2022, The Authors, published by Springer Nature. (B) (i) The schematic diagram of the fabrication of the HA‐Cat‐NCSN hydrogel. (ii) Scheme showing the adhesion of hydrogel on the glass slide. Reproduced with permission.^51^ Copyright 2020, The Authors, published by the American Association for the Advancement of Science. (C) (i) Photos showing the adhesion of the patch on porcine myocardium tissues under stretching, distorting, bending, or immersing underwater. (ii) Self‐healing of the patch. Reproduced with permission.^52^ Copyright 2020, The Authors, published by the American Association for the Advancement of Science.

Aldehyde is another functional group that widely contributes to forming tissue adhesives. The electronegative imbalance between the carbon and oxygen atom imparts the aldehyde with chemical reactivity.^39^, ^40^, ^41^, ^42^, ^43^ The aldehyde can form an imine bond when encountering an amine group based on the Schiff base reactions. For instance, Prince et al. fabricated a nanofibrillar hydrogel (EKGel) for initiating and culturing breast cancer patient‐derived tumor organoids (PDOs). The EKGel was constructed based on the Schiff reaction between the aldehydes on the aldehyde‐modified cellulose nanocrystals and the amines on the gelatin^44^ (Figure 3A). Fan et al. created nature‐derived hydrogels by simply mixing the oxidized alginate and gelatin. The Schiff reactions between aldehydes on the alginate and amines on the gelatin could impart the hydrogel with excellent endoscopic injectability and enable a longer‐lasting submucosal.^45^ Zhang et al. developed a rapid, non‐radical photocoupling reaction to construct a hydrogel adhesive for oral mucosal repair. The hydrogel adhesive consisted of the cyclic o‐nitrobenzyl‐modified hyaluronic acid, which could create three reactive groups under irradiation, including sulfhydryl, nitroso, and aldehyde, of which the sulfhydryl group could crosslink with the nitroso group to form an adhesive rapidly, while the aldehyde group bonded with the protein amino group to achieve tissue adhesion.^46^ In addition, aldehyde can create hemithioacetals when coupled with thiol as well.

Except for NHS and aldehyde, catechol complexes have also been extensively employed to develop tissue adhesive since they are chemically similar to mussel mucoadhesive protein and have a variety of chemical functions.^31^ Catechol is a benzene glycol that consists of a benzene ring and two adjacent hydroxyl groups. An example of a broadly investigated catechol complex is 3,4‐dihydroxyphenylalanine (DOPA). The catechol can react with the amine in various ways after oxidizing to quinone.^47^, ^48^, ^49^, ^50^ HA‐catechol was synthesized by Xu et al., and they demonstrated the fabrication of the pH‐independent hydrogel with superfast gelation time by mixing the HA‐catechol and the reducing thiourea group (NCSN)‐modified HA. The hydrogel exhibited better mechanical strength and could adhere to the stomach and retain for at least 48 h^51^ (Figure 3B). Wang et al. constructed a hybrid patch with anisotropic surface adhesion. Polydopamine (PDA) pregel solution was filled into the PEGDA inverse opal scaffold to form the adhesive layer. The catechol groups on PDA can react with the amines in the tissues^52^ (Figure 3C). Apart from the covalent interaction, noncovalent interactions are used to generate tissue adhesion, such as hydrogen bonds, physical entanglement, and hydrophobic interactions. The synergistic corporation of covalent and noncovalent interactions can attain better tissue adhesion.

## 3 FABRICATION OF THE ADHESIVES

Materials commonly used to prepare tissue adhesives include synthetic materials (cyanoacrylate gum,^53^, ^54^ PEG, and its various derivatives^55^, ^56^, ^57^) and natural materials (fibrin and fibrin‐based materials,^58^ collagen^59^ and gelatin‐based materials,^60^, ^61^, ^62^, ^63^ polysaccharide‐based materials,^64^, ^65^, ^66^, ^67^ etc.). Cyanoacrylate glues, commonly known as universal glues, were the first adhesives used to treat tissue defects, but they are toxic and cause cell necrosis and inflammation. PEG and its derivatives are widely used as tissue adhesives because they are nontoxic, have low immunogenicity, and have adjustable mechanical and biodegradable properties. Several commercial PEG‐based adhesives, such as Coseal™, DuraSeal™, FocalSeal®, and ReSure®, have been tested for in vivo and in vitro tissue adhesion.^3^ For DuraSeal™, it was constructed by mixing the solution of trilysine amine and four‐armed PEG‐NHS. The hydrogel network formed quickly within a few seconds by nucleophilic reactions and the subsequent formation of amide linkages.^68^ However, PEG‐based adhesives are used for bonding minor defects, such as tissue gaps due to cataracts, and their efficacy for more significant damage remains to be investigated.

**FIGURE 4:**
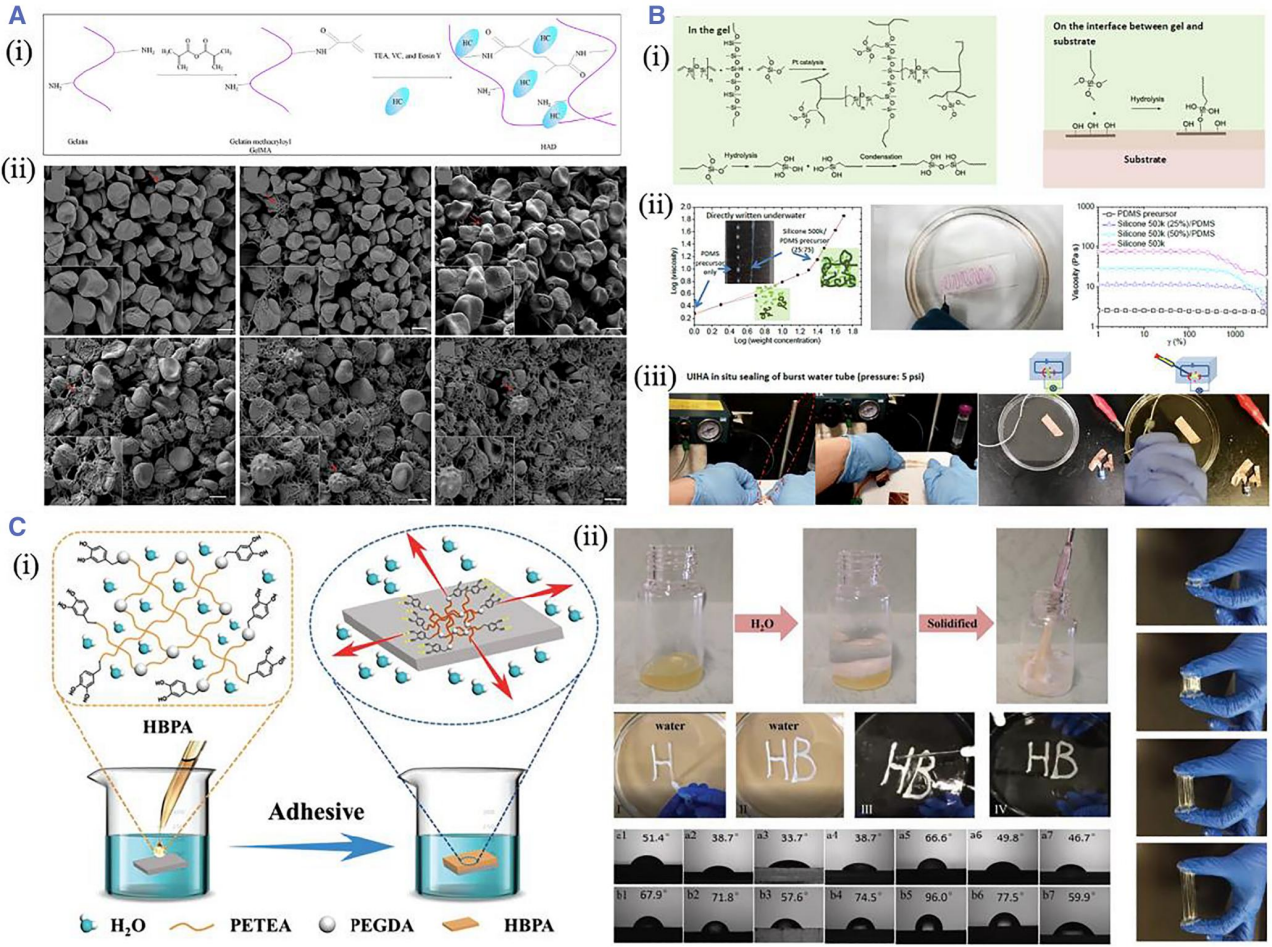
(A) (i) Schematic diagram of photopolymerization of HAD. (ii) SEM images showing the contact of whole blood with GelMA and HAD at different times. Reproduced with permission.^69^ Copyright 2021, The Authors, published by the American Association for the Advancement of Science. (B) (i) The crosslinking and adhesive mechanism of UIHA. (ii) The viscosity curve and underwater writing of UIHA. (iii) Photos showing underwater sealing leakage by UIHA. Reproduced with permission.^77^ Copyright 2022, The Authors, published by the American Association for the Advancement of Science. (C) (i) Schematic representation showing the water‐triggered underwater adhesion and its adhesive mechanism of HBP. (ii) Photos showing the water‐triggered crosslinking and strong adhesion of HBP. Reproduced with permission.^78^ Copyright 2019, John Wiley and Sons. HAD, hemostatic adhesive; HBP, hyperbranched polymer.

The most commonly used adhesives derived from natural materials are fibrin and collagen. Although these adhesives have good biocompatibility and have been commercialized, they have poor mechanical properties and weak tissue adhesion; for animal‐derived materials, there may also be a risk of infection. GelMA, which has improved mechanical properties over gelatin and collagen through chemical modification, has also been used as a tissue adhesive in recent years and has a better effect on tissue matrix repair. But when applied to the clinic, in situ photo‐crosslinking is hazardous for patients with tissue inflammation and photosensitivity.^10^ Inspired by the coagulation activity of snake venom, Guo et al. reported an anti‐hemostatic adhesive (HAD) containing GelMA and hemostatic enzyme (HC) with visible light‐triggered crosslinking. The HAD showed an excellent sealing effect on the severely injured liver and abdominal aorta^69^ (Figure 4A). Yang et al. presented a powerful wet tissue adhesive based on collagen and starch materials (CoSt). CoSt hydrogels are similar to mussel, ivy, and oyster gums in drainage, molecular penetration, enhanced crosslinking, rapid removal of interfacial water, enhanced toughness dissipation, and multiple reversible kinetic effects involved. As a result, they could form strong adhesion and seam‐free seals to injured tissues and make direct contact with tissue fluids or blood, stimulating strong biological interfaces and addressing critical barriers to sutures and commercially available adhesives.^70^ Polysaccharide‐based materials, such as hyaluronic acid, chitosan, and chondroitin sulfate, have good biocompatibility and biodegradability since their molecular structure has natural sugar monomer. Our previous work has demonstrated the fabrication of the hydrogel constructed by mixing the carboxymethyl chitosan and oxidized guar gum. The hydrogel exhibited excellent self‐healing ability and tissue adhesion. Moreover, it could improve wound repair in a rat model of defective skin by promoting cell proliferation, angiogenesis, and collagen secretion.^71^

No matter what chemical strategies are used for tissue adhesion, the interfacial liquid interferes with the adhesion.^72^ Therefore, it is still a complex and challenging task to achieve tissue adhesion in a wet environment.^73^ Researchers have recently investigated and developed different ways to achieve wet adhesion.^74^ It is well recognized that poly (acrylic acid) hydrogel can quickly absorb water since it is highly hydrophilic.^75^ The hydrogel can remove the liquid from the interface and attain strong tissue adhesion when contacting moist tissue through covalent or noncovalent crosslinking. Yuk et al. proposed the DST adhesive composed of polyacrylic acid grafted with NHS and biopolymer. The results from the in vitro and in vivo animal models demonstrated that DST could obtain robust adhesion on a variety of wet tissues within 5 s.^36^ Our previous work constructed a novel bioinspired multilayer patch with anisotropic surface adhesion; the adhesive layer mainly fabricated by the poly (acrylic acid) could achieve super adhesion to wet tissue by absorbing interfacial water and followed by physical and chemical crosslinking. The slippery layer fabricated by liquid paraffin could avoid undesired adhesion to other tissues. The designed novel patches may solve the drawbacks of the traditional surgical suturing and bioadhesives and supply novel patches with anisotropic surface adhesion to the clinic.^76^

Apart from poly (acrylic acid), other adhesives were also developed for wet adhesion. Liu et al. proposed a hydrophobic adhesive (UIHA) that enabled underwater adhesion. The adhesive consisted of polydimethylsiloxane, macromolecular silicone oil, and active silane. The hydrophobic fluids replaced the water at the boundary, forming a gel that adhered to the tissue and obtained superior underwater adhesive strength. It exhibited remarkably more robust underwater shear strength than cyanoacrylate and fibrin glue on porcine skins, showing exceptional water repellency^77^ (Figure 4B). A hyperbranched polymer (HBP) adhesive was constructed by Cui et al. It was composed of a catechol lateral branch with hydrophilic and adhesive properties and a hydrophobic backbone, and its formation was attributed to the Michael addition reaction between polyvinyl monomer and dopamine. The results showed that the hydrophobic chain self‐aggregated and rapidly formed co‐permeates to replace the water molecule at the adhesion interface when contacting with water, triggering enhanced disclosure of the catechol group, resulting in quick and robust adhesion to different materials in a variety of conditions, such as deionized water, phosphate buffered solution, and solutions with various pH values, without the use of any oxidant^78^ (Figure 4C).

## 4 THE APPLICATIONS OF ADHESIVES FOR WOUND CLOSURE

### 4.1 Skin

Skin, as the most prominent tissue in the human body, serves many roles, primarily providing a defensive barrier to external physical forces or chemical products and infectious pathogens and microorganisms.^79^ Trauma or surgical procedures may cause skin injury by impairing its architectural integrity and functions, leading to increased contagious risks.^80^, ^81^ Traditional therapies for treating skin injury are mainly wound dressing and surgical sutures or staples.^82^ Wound dressing can supply a physiologically humid surrounding to the injured area and regulate the exudate.^13^ Nonetheless, most dressings lack adhesive abilities, which need to be used with the assistance of bandages. Suture or staple is widely used for the repair of injured skin.^83^ Although they have high tensile strength and low dehiscence rate, which facilitate wound closure, the drawbacks, such as the high probability of postoperative infection, inflammation, nerve damage, and scar tissue formation, still limit their applications.^84^ Therefore, tissue adhesives have been widely developed for wound closure.^85^, ^86^

**FIGURE 5:**
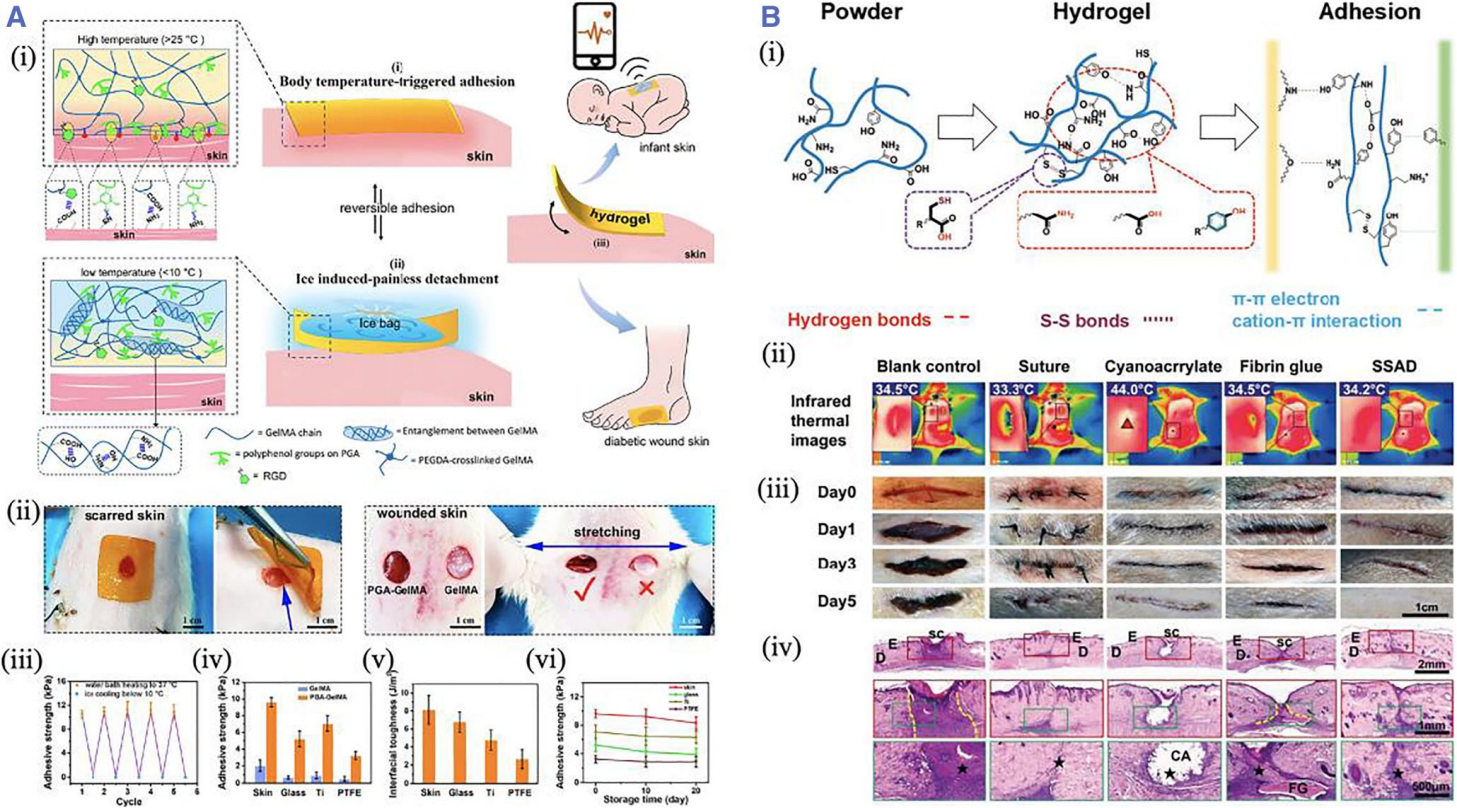
(A) (i) Scheme showing the adhesion and detachment mechanism of the PGA‐GelMA hydrogel. (ii) The adhesion and peeling off the hydrogel on the scarred skin. (iii) The conductivity of the PEDOT‐incorporated hydrogel. (iv–vi) The hydrogel's adhesion strength and interfacial toughness. Reproduced with permission.^87^ Copyright 2022, American Chemical Society. (B) (i) The schematic presentations of the formation and adhesion mechanism of SSAD. (ii) Infrared thermal images. (iii) Photos of the wound. (iv) H&E staining images showing the tissue healing under different conditions. Reproduced with permission.^29^ Copyright 2019, John Wiley and Sons.

A strategy based on polyphenol‐protein complexation was proposed by Jiang et al. to prepare hydrogels with body temperature‐triggered fast adhesion and damage‐free on‐demand peeling. The polyphenol prepolymer (PGA), rich in phenolic and quinone groups, was formed by alkaline oxidative pre‐polymerization. The multiple interactions of polyphenol groups were used in the gelatinization process of GelMA. The PGA‐GelMA hydrogel exhibited mechanical flexibility and high ductility matching the skin tissue and avoiding the damage caused by pulling the hydrogel when peeled on the skin surface. In addition, the PGA‐GelMA hydrogel showed excellent anti‐inflammatory, antioxidant, and anti‐allergic bioactivities, which can effectively avoid skin irritation or allergy caused by conventional skin adhesives in long‐term skin contact^87^ (Figure 5A). Deng et al. first reported a tissue adhesive based on the skin secretion of *Andrias davidianus* (SSAD) to repair damaged skin. SSAD bagel has high adhesive strength, maintains flexibility at the bonding interface, promotes skin wound healing, and degrades entirely within 3 weeks. The adhesion mechanism in the subcutaneous adipose tissue of SSAD is attributed to the hydrophobic groups of some amino acids of SSAD interacting with hydrophobic fats in the subcutaneous adipose tissue via hydrophobic forces. In addition, the cationic residue of the lysine moiety could substitute hydrated cation out of the tissue interface in a wet environment, thereby resulting in the adhesion of the phenyl groups of phenylalanine to subcutaneous tissues through cation‐π interactions. Thus, the combination of hydrophobic interactions, cation‐π interactions, and the intrinsic hydrogen bonds between the hydroxyl/amido moieties of the hydrogel and carboxyl moieties of fatty acid may promote firm adhesion to adipose tissues^29^ (Figure 5B).

### 4.2 Cornea

The cornea is a vital tissue in the visual system. Trauma, infection, autoimmune diseases, and burns are the primary triggers of corneal scarring and damage, resulting in it being one of the leading blindness‐causing diseases worldwide.^7^, ^88^, ^89^, ^90^, ^91^ Currently, there are two types of standard corneal patches according to the grafting method: suture‐type patches that need to be fixed to the injured area using surgical sutures and noninvasive adhesive‐type patches that can be used as adhesives themselves and adhered directly to the injured area.^76^, ^92^ However, the surgical suturing process damages the cornea, and the residual sutures may cause inflammation and vascularization. Therefore, adhesive materials have emerged as a progressive therapy for healing corneal injury.^26^ Generally, materials utilized to engineer corneal replacement should have a structure and physiochemical properties similar to those of the natural cornea. The desired material to promote corneal restoration should be biocompatible and biodegradable, mechanically stable, highly transparent, highly adhesive to the cornea, and capable of supporting the growth of cells and regeneration of tissues.^93^

**FIGURE 6:**
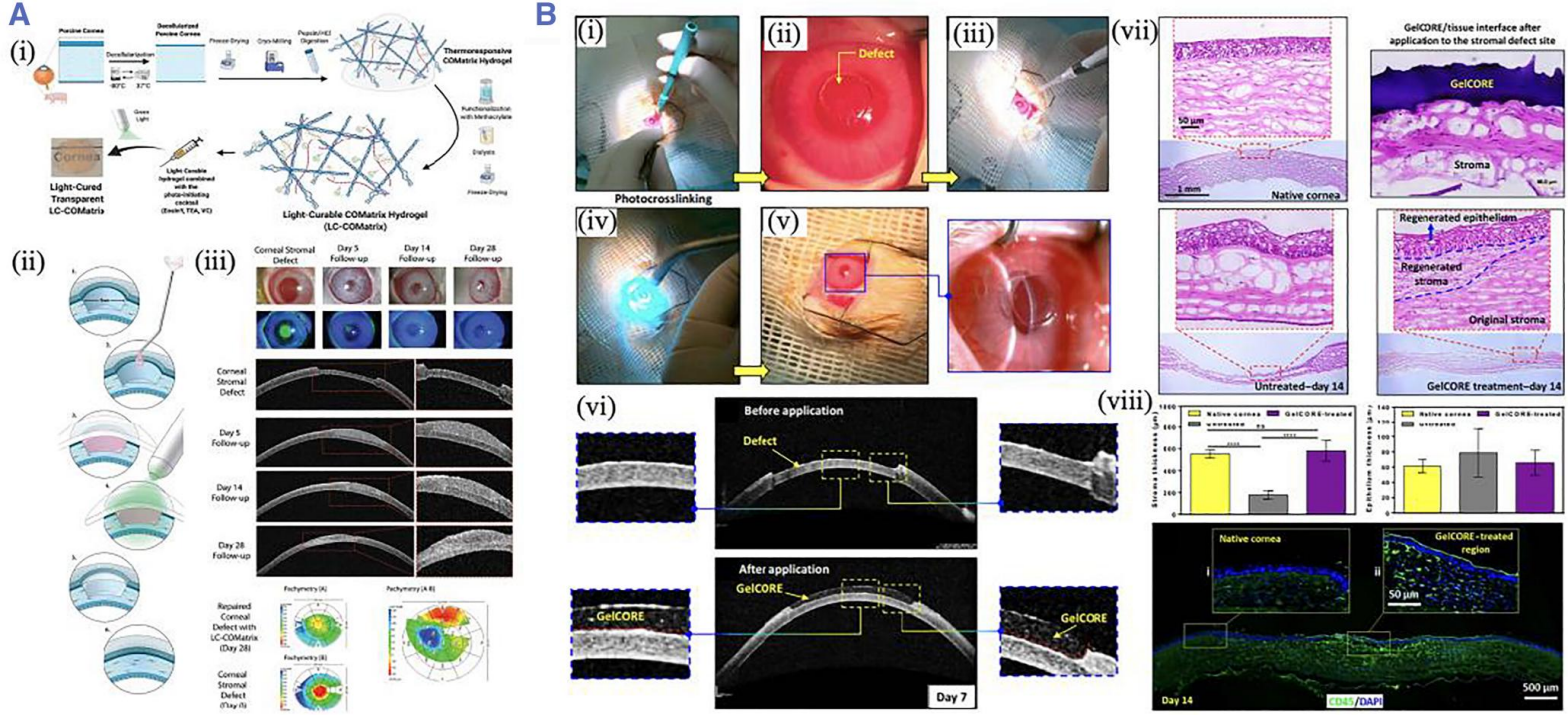
(A) (i) The schematic diagram of the fabrication of LC‐COMatrix hydrogel. (ii) Scheme showing the animal experiment process. (iii) The slit‐lamp, OCT, and pachymetry difference map images. Reproduced with permission.^94^ Copyright 2022, John Wiley and Sons. (B) (i–vi) In vivo performance of GelCORE bioadhesive into rabbit corneal defect. (vii) HE staining images of the native cornea and defective cornea filled with GelCORE. (viii) Thickness of epithelium and stroma after the operation and immunofluorescent images. Reproduced with permission.^95^ Copyright 2019, The Authors, published by the American Association for the Advancement of Science.

Yazdanpanah et al. introduced a light‐cured corneal matrix (LC‐COMatrix), which was synthesized from the decellularized porcine cornea containing undenatured collagen. The LC‐COMatrix was multifunctional and had an appropriate swelling ratio, biodegradability, and viscosity to apparently improve the mechanical property, stability, and adhesion of the hydrogel. The LC‐COMatrix can adhere firmly to the human cornea and effectively occlude the corneal perforation. The in vivo studies also demonstrated that the LC‐COMatrix could seal corneal perforation and replace cornea stromal defects in a rabbit model^94^ (Figure 6A). Sani et al. fabricated GelMA‐based hydrogels (GelCORE) for the sutureless repair of corneal injuries. The physiochemical abilities of GelCORE can be well regulated by changing the polymer concentrations and photo‐crosslinking time. GelCORE demonstrated superior tissue adhesive strength compared to commercially available adhesives. In vivo experiments indicated that GelCORE can effectively adhere to the defected cornea and induce stromal regeneration and re‐epithelialization^95^ (Figure 6B).

### 4.3 Gastrointestinal tissue

Apart from skin and cornea, tissue adhesives have also been widely used in gastrointestinal (GI) tissues. The primary function of GI tissue is to transport, digest, and absorb food for the body. GI tissue surgery usually needs the anastomosis between two discrete tissues for the restoration of GI continence.^96^ GI surgeries may induce severe complications, such as anastomotic dehiscence, which is primarily destructive and can lead to leaky luminal components and hemorrhage.^96^, ^97^ A variety of technics, such as sutures and staples, are widely employed to sustain anastomotic conservation. However, anastomotic leakage can still happen in as high as 23% of cases, and low colorectal and coloanal anastomoses can lead to increased mortality.^98^ In recent years, tissue adhesives have been developed to enhance the lines of sutures and staples for the prevention of leakage.^27^, ^99^, ^100^, ^101^ Various materials are employed as GI tissue adhesives, such as fibrin, albumin‐based, PEG‐based, and gelatin‐based adhesives. Fibrin is a potent sealing agent with excellent biocompatibility in GI procedures.^102^, ^103^ Still, the ability to improve wound healing is limited, and it may inhibit bacterial phagocytosis by immune cells. Although albumin‐based adhesives can offer a strong sealing for anastomosis, the high stiffness and poisonous by‐product can be risky.

**FIGURE 7:**
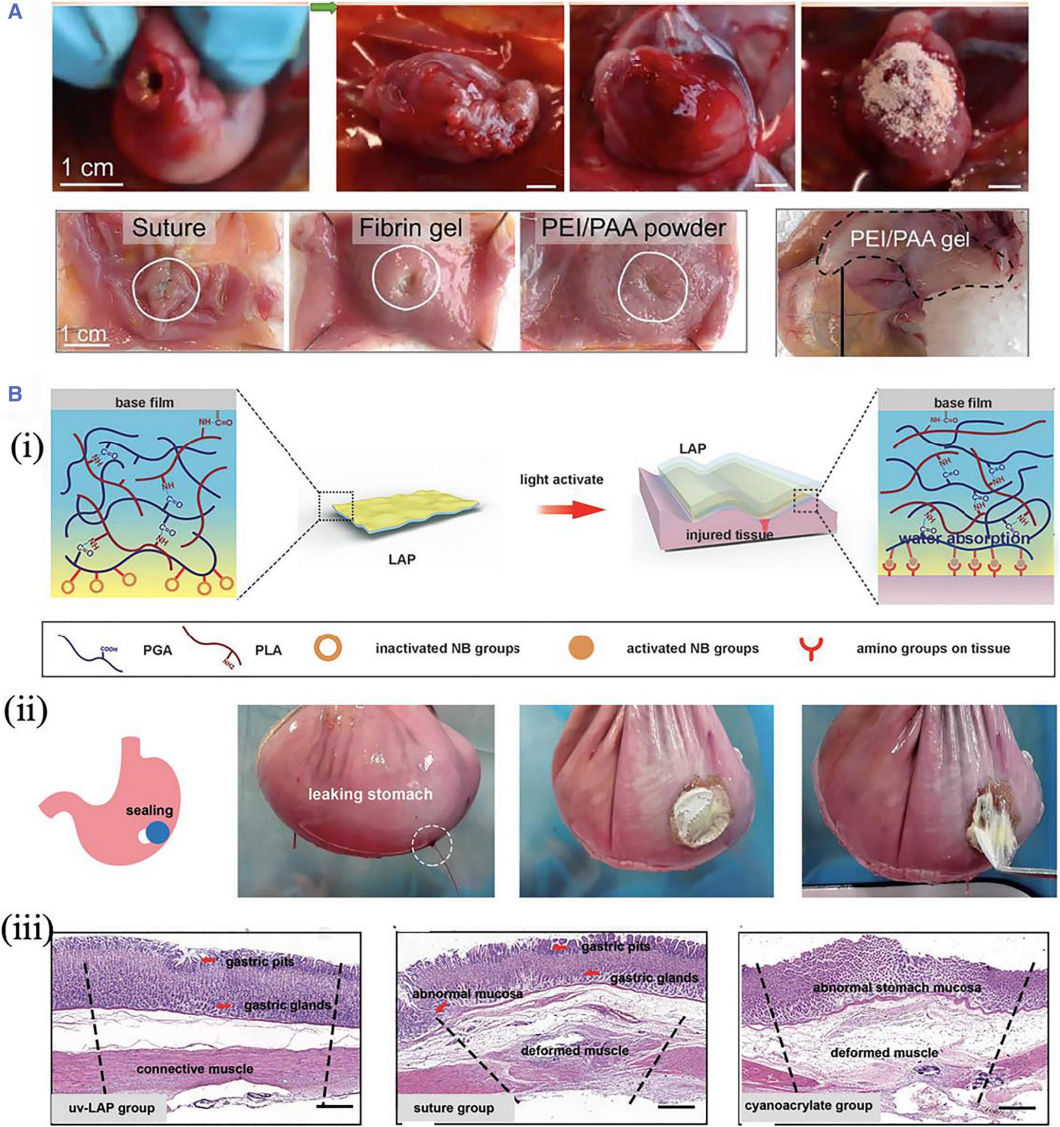
(A) Photos showing the surgical procedure for sealing gastric perforation. Reproduced with permission.^104^ Copyright 2021, The Authors, published by the American Association for the Advancement of Science. (B) (i) Schematic diagram presenting the design of LAP. (ii) Photographs of the LAP adhesion on the porcine stomach. (iii) The histological images of the stomach treated under different conditions. Reproduced under terms of the CC‐BY license.^105^ Copyright 2022, The Authors, published by John Wiley and Sons.

Researchers presented a polyethyleneimine and polyacrylic acid (PEI/PAA) powder with self‐gel and adhesion properties that could upscale interfacial water in situ to construct a physically crosslinked hydrogel within a few seconds because of the strong physical interactions between the materials. In addition, the physically crosslinked materials could penetrate the substrate polymer networks to improve wet adhesion. The PEI/PAA powder surface deposition can seal the injured pig stomach and intestine despite their irregular and highly mechanically challenging surfaces. The researchers further demonstrated that PEI/PAA powder was an effective sealer for improving the healing of gastric perforation in rats. PEI/PAA powder has robust wet adhesion, good compatibility, adaptability to complicated sites, and ease of synthesis, making it a promising bioadhesive with promising applications^104^ (Figure 7A). McTiernan, C.D. et al. proposed an adhesive patch triggered by light (LAP) for sutureless sealing and healing visceral wounds with opening defects. LAP was composed of a peptide hydrogel with high water absorption, an adhesive layer decorated with a butyramide (NB) group, and a basal membrane constructed by poly (l‐lactic acid) (PLLA) to improve mechanical stability. Light‐activated LAP has been shown to rapidly and firmly close multi‐visceral open wounds with only 15 s pressure on the defect. In vivo studies have demonstrated that LAP had good biodegradability and provided an immunological microenvironment favorable for angiogenesis and tissue regeneration. In a rabbit gastric perforation model, LAP can be used for sutureless wound closure and total gastric repair. The progress made in this study will demonstrate next‐generation adhesive patches with mechanical abilities and macrophage modulation capabilities^105^ (Figure 7B).

## 5 CONCLUSION AND PERSPECTIVES

In summary, this review mainly discussed tissue adhesives for wound closure. In the first part, we introduced the fundamental design requirements for the adhesives. The nature of tissues and the functional chemical groups on the tissue surface was presented. The most widely used reactive groups for the adhesives, including the covalent interaction, such as NHS, aldehyde, and catechol, and noncovalent interactions, such as hydrogen bond, physical entanglement, and hydrophobic interactions, were then discussed. In the second part, we summarized the fabrication and significant recent advances of several tissue adhesives, including synthetic and natural materials‐derived adhesives. Specifically, the adhesives fabricated for wet adhesion were emphasized and introduced. In the third part, we discussed the application value of the adhesives in skin healing, corneal patch, and gastrointestinal tissues with examples that have achieved higher performances and advanced functionalities.

Although academic and industrial research has demonstrated fascinating achievements, tissue adhesives still have several limitations and significant opportunities. First, the mechanical properties and adhesion strength of the existing adhesives are not sufficient and need further enhancement. The poor performance of the adhesives makes it impossible to completely substitute for traditional surgical sutures, but only as an adjunctive treatment therapy. As a result, commercialized tissue adhesives have shown limited success in the repair of tissues. Significant advances in the engineering of adhesives, which can improve the strength of adhesives, potentially offer the opportunity to overcome this discrepancy. Second, the assembly of multi‐functions in one adhesive should be emphasized and enhanced. Apart from the essential functions, such as antibacterial, anti‐inflammatory, and pro‐angiogenic properties, health monitoring and diagnosis should also be developed. Numerous efforts are underway to amplify detectable life signals to facilitate improved monitoring and diagnosis, for example, by incorporating biological sensing capabilities into the adhesives. Third, the clinical translation of most adhesives still meets significant challenges with low translational efficiency. Although extensive research results have demonstrated the design and fabrication of tissue‐specific adhesives with superior performance, most adhesives are only verified in animal models. They are far away from the next stage of the clinical trial. We believe the ongoing interdisciplinary and cooperative endeavors will provide novel ideas, materials, and approaches for tissue adhesives.

## AUTHOR CONTRIBUTIONS

Zhou Liu, Tiantian Kong, and Huan Wang conceived the topic of the manuscript. Bin Kong wrote the manuscript. Cheng Qi contributed to the revision of the manuscript.

## CONFLICT OF INTEREST STATEMENT

The authors declare no competing financial interests.
